# Reasoning on the Autism Spectrum: A Dual Process Theory Account

**DOI:** 10.1007/s10803-016-2742-4

**Published:** 2016-03-09

**Authors:** Mark Brosnan, Marcus Lewton, Chris Ashwin

**Affiliations:** Department of Psychology, University of Bath, Bath, BA2 7AY UK

**Keywords:** Autism, Reasoning, Intuition, Deliberation

## Abstract

Dual process theory proposes two distinct reasoning processes in humans, an intuitive style that is rapid and automatic and a deliberative style that is more effortful. However, no study to date has specifically examined these reasoning styles in relation to the autism spectrum. The present studies investigated deliberative and intuitive reasoning profiles in: (1) a non-clinical sample from the general population with varying degrees of autism traits (*n* = 95), and (2) males diagnosed with ASD (*n* = 17) versus comparisons (*n* = 18). Taken together, the results suggest reasoning on the autism spectrum is compatible with the processes proposed by Dual Process Theory and that higher autism traits and ASD are characterised by a consistent bias towards deliberative reasoning (and potentially away from intuition).

## Introduction

Autism Spectrum Disorder (ASD) is a neurodevelopmental disorder of unknown underlying etiology characterised by persistent deficits in social communication and social interaction combined with restricted, repetitive patterns of behaviour, interests, or activities (APA 2013) with prevalence estimates up to one in 68 children (CDC 2015). A continuum of autism traits extend throughout the general population until they become clinically significant under ASD diagnostic criteria to form part of an ‘autism spectrum’ of autistic presentation (Constantino and Todd 2003; Plomin et al. 2009; Posserud et al. 2006; Wing 1988; see Ruzich et al. 2015, for systematic review). Ruzich et al. report that, within the general population, males have significantly higher levels of autism traits than females, and those with ASD have significantly higher autism traits than males from the general population (with no sex differences in autism traits within the ASD population).

Reasoning and decision making are core human capabilities that enable effective participation within society, yet have received relatively little attention within the autism spectrum literature. Reasoning in a manner which is normatively logical and subsequent rational decision making are specialised higher cognitive functions attributed to common processing mechanisms (see Evans and Stanovich 2013). Luke et al. (2012) identified three core features of reasoning and decision making that were particularly problematic for people with ASD using a self-report methodology. Decisions were difficult to make for people with ASD if they involved talking to others; involved a change in routine; or if the decision has to be made quickly. Whilst difficulties talking with others and changes in routine reflect the core diagnostic features of ASD (respectively), the difficulty with rapid decision making for those with ASD does not, and may provide added insight into reasoning on the autism spectrum.

A more deliberative approach to reasoning has been proposed to characterise people with ASD compared to the general population. For example, De Martino et al. (2008) report that people with ASD reason in a more logically consistent manner than matched controls. People with ASD also request more information prior to making a decision upon a probabilistic reasoning task compared to controls, a style of reasoning that has been termed a ‘circumspect reasoning bias’ (Brosnan et al. 2014a). Similarly, those from the general population who self-reported being higher in autism traits also require more information prior to making decisions when compared to those lower in autism traits (Brosnan et al. 2013). This behavioural data is consistent with self-reports from people with ASD about their difficulties with rapid decision-making and reasoning and a preference for more deliberation (Luke et al. 2012). Contrasting rapid decision-making/reasoning processes with deliberative decision-making/reasoning processes forms the basis of Dual Process Theory. When considering human reasoning, Dual Process Theory has been a dominant model within cognitive psychology for almost 50 years (Evans and Frankish 2009). The dual processes are referred to as Type 1 and Type 2 and will be referred to as intuition and deliberation (respectively) for convenience. Intuition involves rapid, effortless, parallel, non-conscious processing that is independent of working memory and cognitive ability. Deliberation, on the other hand, involves slower, effortful, sequential, conscious processing and is heavily dependent on working memory and related to individual differences in cognitive ability (see Evans 2011; Evans and Stanovich 2013; Kahneman 2011; Stanovich and West 2000, 2008; for reviews; see Keren and Schul 2009 for critique; see Kruglanski and Gigerenzer 2011 for an alternative view).

Within Dual Process Theory, rapid autonomous processes (‘intuitive reasoning’) are assumed to yield default responses unless intervened upon by distinctive higher order reasoning processes (‘deliberative reasoning’). Intuitive reasoning preceding deliberative reasoning is known as the default-interventionist position (see Evans and Stanovich 2013; Kahneman 2011). One of the most widely used behavioural assessments of intuition and deliberation is the Cognitive Reflections Test (CRT: Frederick 2005). The CRT comprises of three reasoning questions that have both an intuitive (incorrect) and deliberative (correct) response. A majority of intuitive responses are typically provided for the CRT questions (Frederick 2005), which is theorised to reflect the output from initial intuitive reasoning which has not been over-ridden by deliberative reasoning. The over-riding of initial intuitive reasoning by subsequent deliberative reasoning is demonstrated by achieving the correct answer. In support of this, experimental manipulations designed to encourage participants to engage in deliberative reasoning reduces intuitive responses (Evans and Curtis-Holmes 2005).

Intuitive reasoning is also argued to be evidenced by ‘the framing effect’ (Tversky and Kahneman 1974) in which logical decision making is influenced by the context of the reasoning task. De Martino et al. (2008: 10746) report a decreased susceptibility to the framing effect in people with ASD, who demonstrate an ‘unusual enhancement in logical consistency’. Within the context of Dual Process Theory, De Martino et al. (2008) hypothesise that individuals with ASD have an increased tendency towards deliberation, attributable to impairment within intuitive reasoning systems. This is consistent with Klin and Volkmar (1997: 102) observations of people with Asperger’s Syndrome as having ‘a deficient intuition and lack of spontaneous adaptation’ (see Allman et al. 2005). Klin et al. (2003) propose an embodied cognition ‘Enactive Mind’ approach for understanding social adaptation, which is argued to have important temporal constraints. Social adaptation is reasoned to require the processing of salient stimuli based upon split-second environmental demands with moment-by-moment disregard of stimuli perceived as irrelevant. Under this approach, people with ASD do not reflect the typical processing bias towards socially relevant stimuli.

In addition to the CRT behavioural measure, the propensity to engage in intuitive and deliberative reasoning can be assessed through self-report. The Rational-Experiential Inventory (REI) is a widely used measure of intuition and deliberation (Epstein et al. 1996). The Rational component is based upon a ‘need for cognition’ (Cacioppo and Petty 1982) which measures engagement in, and enjoyment of, cognitive activities. The Experiential component was developed to measure engagement and confidence in one’s intuitive abilities and is termed ‘faith in intuition’ (Epstein et al. 1996; Pacini and Epstein 1999). Epstein et al. argue that these two information processing styles are independent of one another, such that one can be high or low in either or both dimension.

Deliberative responses on the CRT have been found to positively correlate with REI self-reported deliberation and negatively with REI self-reported intuition. Additionally, intuitive responses on the CRT have been found to positively correlate with REI self-reported intuition and negatively with REI self-reported deliberation (Pennycook et al. 2015). However, other studies have only reported the positive relationship between deliberation on the CRT and REI (Liberali et al. 2012; Thoma et al. 2015). Thus, the variability between self-reported preference for intuition and behavioural intuition needs to be borne in mind. Freeman et al. (2012) found that combinations of high and low intuition with high and low deliberation, as measured by the REI, best predicted clinically relevant traits (schizotypy) in a general non-clinical population. The literature above would suggest that autism traits in a general population would best be predicted by a combination of high deliberative and low intuitive reasoning styles.

The aim of the present research was to investigate intuitive and deliberative reasoning across two studies that focus on the autism spectrum; one involving a non-clinical sample, and the other involving a clinical sample. As a continuum of autism traits is proposed to extend throughout the (general and ASD) population, the relationship with reasoning was examined in relation to relatively higher and lower levels of autism traits. In the non-clinical sample it was predicted that higher levels of autism traits would relate to a profile characterised by greater deliberative and reduced intuitive reasoning. Study 2 compared self-report and behavioural measures of intuitive and deliberative reasoning between people with and without ASD, and it was predicted that the ASD group would show a more deliberative and less intuitive profile than the comparison group.

## Study 1

### Methods

#### Participants

Participants were 95 undergraduate students from a range of disciplines (43 male, 52 female) aged 18–31 years old (mean = 21.0, SD = 4.01, see Table 1) recruited at the University of Bath. All participants were native English speakers, and no participant reported a diagnosis of a mental health condition. Participants were rewarded with either course credit for their participation or received £5.00. The research was approved by the Psychology Departmental Research Ethics Committee at the University of Bath, which implements the ethical guidelines of the British Psychological Society.

**Table 1:** Mean scores for age, autism traits (AQ) and reasoning (*n* = 95)

Measure	Mean (SD)	Min	Max	Male mean (SD) *n* = 43	Female mean (SD) *n* = 52
Age	21.00 (4.01)	18.00	31.00	21.14 (3.97)	20.21 (3.02)
Total AQ	20.00 (11.53)	4.00	45.00	18.60 (11.59)	19.35 (9.44)
Mean intuition	3.20 (.62)	1.75	4.10	3.15 (.70)	3.23 (.56)
Mean deliberation	3.51 (.61)	1.80	4.85	3.60 (.53)	3.44 (.66)

#### Procedure

Autism traits were assessed using the AQ (Baron-Cohen et al. 2001a, b), which is a self-report measurement that is used to identify autism traits in clinical and non-clinical adult populations. Participants rated their level of agreement with 50 items (e.g. “I enjoy doing things spontaneously”) on a 4-point Likert scale ranging from 0 (‘definitely disagree’) to 4 (definitely agree). A response in the direction of autism characteristics is scored as 1, while a response in the opposite direction is scored as 0. This results in scores ranging from 0 to 50. The AQ in the present study had a high level of internal consistency, with a Cronbach’s alpha of .88.

Reasoning processes were assessed using the Rational Experiential Inventory (REI; Pacini and Epstein 1999). The REI measures a participant’s preference for both intuitive (experiential) and deliberative (rational) reasoning. The REI is a 40 item questionnaire, containing 20 items that assess intuitive reasoning and 20 items that assess deliberative reasoning. Examples from the intuitive scale include, “I trust my initial feelings about people” and “I often go on my instincts when deciding on a course of action”. Examples of items from the rational scale include, “I have a logical mind” and “I enjoy solving problems that require hard thinking”. Respondents score each item on a 5-point scale, from 1 = completely false to 5 = completely true. Mean scores for each subscale can therefore range from 1 to 5 for each reasoning style. In the present study the Cronbach’s alpha for the experiential-intuitive scale was .91, and for the rational-deliberative scale it was .88.

### Results

The total scores for AQ, intuition and deliberation are displayed in Table 1. There were no significant sex differences for self-reported autism traits, intuition or deliberation (all *p* > .05). Following Freeman et al. (2012), a median-spilt method was used across both intuitive and deliberative scores to divide the participants into one of four groups: namely (1) high intuition/high deliberation; (2) low intuition/low deliberation; (3) high intuition/low deliberation; or (4) low intuition/high deliberation. (Freeman et al. 2012). The numbers of males and females in each group were: (1) 9:11; (2) 9:14; (3) 13:14; and (4) 12:13; which did not differ significantly (chi = .516, *p* ≥ .05).

**Fig. 1:**
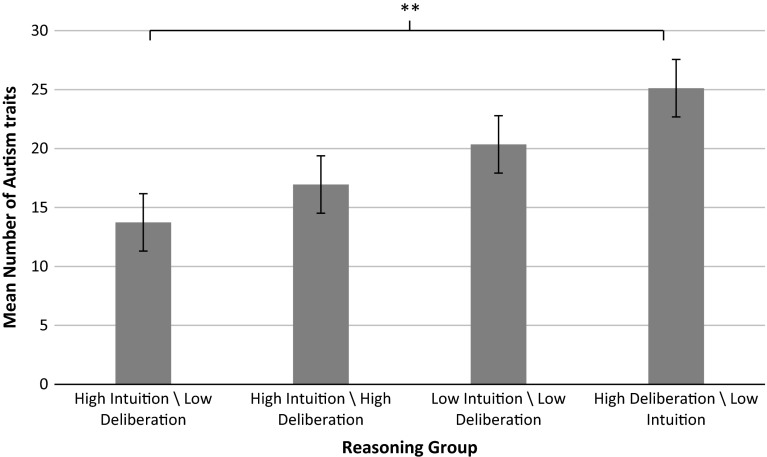
Mean Autism traits by the interaction of intuitive and deliberative reasoning process. **p < .01

A one-way ANOVA revealed that the high deliberation/low intuition group had a significantly higher AQ total than the low deliberation/high intuition group (*F* (3, 91) = 19.87, *p* < .01; all other comparisons *p* > .05; see Fig. 1).

### Discussion of Study 1

Study 1 examined the extent to which autism traits in a non-clinical population were associated with self-reported preferences for more deliberative over intuitive reasoning. Results found higher autism traits in those with a profile consisting of low intuitive and high deliberative reasoning, compared to those with a high intuitive and low deliberative profile of reasoning. Those with comparable levels of self-reported intuitive and deliberative reasoning, whether the levels were both high or low, did not differ from each other in their levels of autism traits. Consistent with the hypothesis, higher autism traits were associated with a combination of greater deliberative and less intuitive reasoning styles. This is consistent with the clinical literature, where ASD is associated with a more logical and circumspect reasoning bias (Brosnan et al. 2014a; De Martino et al. 2008).

Interestingly, the present findings are the opposite results to that reported by Freeman et al. (2012) for schizotypy traits in the general population, where higher levels of schizotypy traits were associated with high levels of intuition combined with low levels of deliberation. This opposing reasoning profile is consistent with the diametrical model of Crespi and Badcock (2008), who propose that ASD and schizotypy represent opposing poles of a cognitive continuum. Thus a bias towards deliberative reasoning and away from intuitive reasoning may characterise reasoning associated with higher autism traits (with the opposite pattern characteristic of higher schizotypy traits). Whilst this analysis is useful for comparative purposes, it is not intended to reify these groupings. Figure 1 highlights that the two reasoning style combinations containing high intuition were associated with the lower levels of autism traits and the two reasoning style combinations containing low intuition were associated with higher levels of autism traits. Study 1 therefore provided initial support for autism traits within the general population being relevant to Dual Process Theory. However, the REI provides a self-reported reasoning preference rather than an assessment of reasoning behaviour, although previous research has suggested a correlation between the two (Liberali et al. 2012; Pennycook et al. 2015; Thoma et al. 2015). Study Two extended the investigation to a clinical population with Autism Spectrum Disorder and included a behavioural measure of intuition and deliberation, the CRT (Frederick 2005).

## Study 2

### Methods

#### Participants

Participants were 17 males with ASD and 18 typically developing (TD) males without ASD who served as the comparison group. The ASD group had a mean age of 18.4 years (range 17–21; SD = 1.3) and the TD group had a mean age of 19.5 years (range 16–21; SD = 1.9; the difference in age between groups did not reach statistical significance (*t*(31) = 1.94, ns; see Table 2). The research was approved by the Psychology Departmental Research Ethics Committee at the University of Bath which implements the ethical guidelines of the British Psychological Society.

**Table 2:** Means (and SD) for ASD and TD groups for demographics and dual process self-report and behavioural measures

Demographics and variables	Group		*t*	Cohen’s *d* [95 %CI]
	ASD (*n* = 17)	TD (*n* = 18)		
Age	18.4 (1.3)	19.5 (1.9)	1.94	
CRT-intuition	.71 (.92)	1.41 (1.1)	2.07*	.69 [.00–1.40]
CRT-deliberation	2.00 (.87)	1.12 (.99)	2.76**	.94 [.24–1.52]
REI-intuition	2.84 (.89)	3.80 (.53)	3.67***	1.40 [.42–1.50]
REI-deliberation	4.09 (.70)	3.63 (.71)	1.70	.65 [−.09 to 1.01]

Cohen’s* d* effect size, with 95 % Confidence Intervals

* *p* < .05, ** *p* < .01, *** *p* < .001. 6 participants with ASD did not complete the REI

The ASD Group comprised of participants attending a University Summer School for students on the autism spectrum focussed on providing an insight into university life. On application to the summer school, students provided evidence of clinic diagnosis of ASD using international criteria (DSM-IV, APA 1994; ICD-10, WHO 1992) by a qualified professional. ASD diagnosis was then confirmed using the Social Communication Questionnaire (SCQ-Lifetime; Rutter et al. 2003), a 40 item parent report measure. The SCQ is a dimensional measure of ASD symptomatology, with a sensitivity of .92 and specificity of .62 (Witwer and LeCavalier 2008). In addition, the Ritvo Autism Asperger Diagnostic Scale-Revised (RAADS-r; Ritvo et al. 2011) was also utilised, which is an 80 item self-report measure assessing four symptom areas: language, social relatedness, sensory-motor, circumscribed interests. The RAADS-r has a sensitivity of .97 and specificity of 1 (Ritvo et al. 2011). Scores on both measures were significantly above the clinical cut-offs (Mean SCQ score = 19.75, SD = 5.07, range 11–27; *t*(15) = 3.75, *p* = .002; and mean RAADS-R score = 113, SD = 21.77, range 65–140; *t*(16) = 9.09, *p* < .001). The TD group was an opportunity sample of male students commencing their first year at the same university. TD participants completed the AQ10, which is a ten item version of the AQ which can be used for screening purposes. A cut off of 6 or greater indicates a referral to diagnostic services may be appropriate (Allison et al. 2012). Scores ranged from 0 to 4, with a mean of 2.1 (SD = 1.1) indicating the TD group did not have a screening score warranting investigation for an ASD.

#### Procedure

The Rational-Experiential Inventory-Short (REI-S: Epstein et al. 1996) was developed as a short version of the questionnaire used in Study One which contains 10 items, equally divided between intuitive and deliberative subscales. The short version was used as time was limited at the Autism Summer School, however 6 members of the ASD group still did not complete the REI-S.

The Cognitive Reflection Task (CRT: Frederick 2005) is a widely used 3-item performance measure of intuition and deliberation. Each question has a potentially intuitive and deliberative answer, as well as the potential for wrong answers. Scores can therefore range from 0 to 3 for each subscale. (Note, the intuitive response is a wrong answer). An example item is: ‘A bat and ball cost £1.10 in total. The bat costs £1 more than the ball. How much does the ball cost?’ The intuitive answer is 10 pence (cents in USA version) and the deliberative answer (which is correct) is 5 pence. All other responses are considered wrong. Planned *t* tests compared between group differences on the REI and CRT, in addition to one-sample *t* tests comparing the CRT to expected means.

### Results

**Fig. 2:**
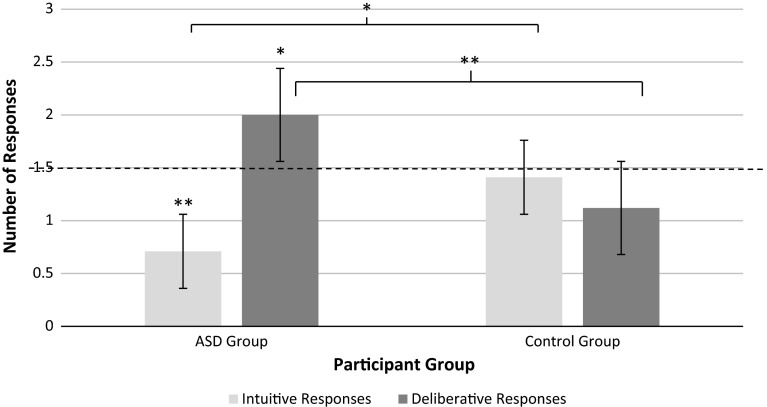
Mean group differences between intuitive and deliberative reasoning responses on the CRT by Group. *Note* *Dotted line* represents mid-point of potential scores. Significant difference from this represented by **p < .01, *p < .05

The means for each group are highlighted in Table 2. Independent-samples *t* tests showed that the ASD group provided more deliberative and less intuitive responses than the TD group on the behavioural CRT measure (see Fig. 2). Cohen’s *d* analysis indicated medium to large e effect sizes. Since a mean of 1.5 represents the middle neutral point of responding between intuitive and deliberative responses on the CRT, one-sample *t* tests were carried out for the CRT scores of each group to see if they were significantly responding towards one style or the other. Results highlighted that the TD group means did not significantly differ from the middle neutral value (both *p* > .05). For the ASD group, both the number of intuitive (*t*(16) = 3.56, *p* < .01) and deliberative responses (*t*(16) = 2.38, *p* < .05) differed from the middle value 1.5; see Fig. 2). 8 incorrect responses were provided by the TD group and 5 by the ASD group which were not analysed (this number did not significantly differ between groups (*t*(32) = .93, ns).

**Fig. 3:**
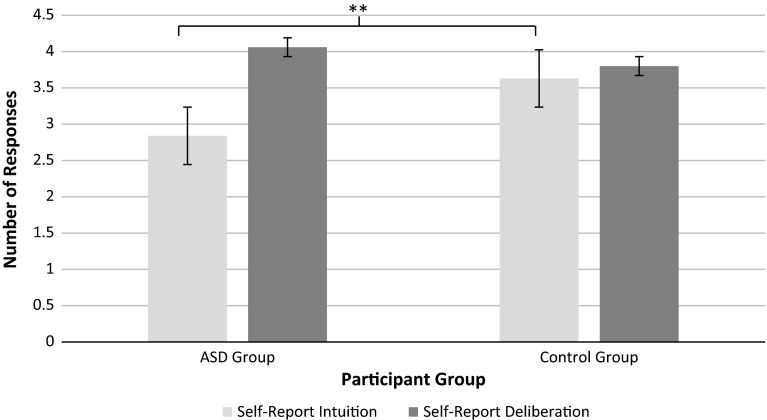
Intuitive and deliberative responses to the REI by Group

Further *t* tests also showed the ASD group self-reported significantly lower levels of intuition than the TD group using the REI (*t*(27) = 3.67, *p* < .001). There was also a trend for the ASD group to self-report higher levels of deliberation than the TD group (*t*(27) = 1.7, *p* < .1), see Fig. 3.

### Discussion of Study 2

Study 2 demonstrated, for the first time, that young male adults with ASD responded on the CRT in a less intuitive and more deliberative manner. The task is not purely ipsative as it is also possible to make errors. However, providing an intuitive answer does necessitate that a deliberative answer is not provided. The REI (short) is not ipsative (in that one could self-report being high in both), and again lower intuition was evidenced in the ASD group along with a trend towards higher deliberation. Taken together, the data are consistent with a Dual Process Theory account of ASD as a bias away from intuitive reasoning and towards deliberative reasoning (Brosnan et al. 2014a; De Martino et al. 2008). The term ‘bias’ is appropriate as those with ASD tended to respond intuitively half as often as TD participants and respond deliberatively twice as often as TD participants. This is clearly different to *only* responding in a deliberative manner. It may suggest intuitive processes can be employed by those with ASD, though just not as frequently or easily as TD participants. Dual Process Theory proposes that intuitive processes represent default responses unless intervened upon by deliberative processes (Evans and Stanovich 2013). De Martino et al. (2008) hypothesise that a logical reasoning bias in ASD is attributable to impairment within the intuitive reasoning mechanisms and the evidence of limited intuitive responding by those with ASD in the present study is consistent with this.

## General Discussion

Two studies explored the relationship between Dual Process Theory of human cognition and reasoning on the autism spectrum. People with high autism traits and those diagnosed with ASD showed a pattern of having a combination of lower intuitive and greater deliberative reasoning styles. Both those with high autism traits and those with a diagnosis of ASD consistently responded less intuitively and more deliberatively, when compared to those with low autism traits and without a diagnosis of ASD, on behavioural and self-report assessments of reasoning. Taken together, the results suggest that Dual Process Theory provides a useful framework for considering the strengths and weaknesses in reasoning on the autism spectrum. De Martino et al. hypothesised that those with ASD may have compromised intuitive reasoning, based upon those with ASD demonstrating enhanced logical consistency. Consistent with this, this is the first study to demonstrate enhanced deliberative responding explicitly in those with ASD and those with high autism traits, which is consistent with enhanced logical consistency or a circumspect reasoning bias (Brosnan et al. 2014a; De Martino et al. 2008).

Within the default-interventionist perspective of dual process theory (see Evans and Stanovich 2013; Kahneman 2011), intuitive processes are assumed to yield default responses unless intervened upon by distinctive higher order reasoning processes (deliberative). There are at least three potential explanations for the present findings: (1) those with ASD have impaired intuitive mechanisms and consequently deliberative reasoning is dominant; (2) intuitive mechanisms are intact but dominated by deliberative reasoning; or (3) Intuitive mechanisms are intact in different contexts but this context triggers deliberative reasoning in those with higher levels of autistic traits. Under this Dual Process model, a slower, effortful, sequential, deliberative reasoning style would be dominant in ASD as a consequence of impairment in rapid, effortless, parallel intuitive mechanism (De Martino et al. 2008), though the possibility remains that this may be due to dominant deliberative reasoning (possibly within the context of the task). Either way, higher autism traits would be associated with a propensity to engage in deliberative reasoning rather than being associated with deliberative reasoning abilities per se (e.g. a greater preference for deliberative reasoning within an individual does not necessarily entail greater deliberative skills within that individual). This is consistent with Luke et al. (2012) who found that those with ASD did not self-report a greater reliance upon a rational/deliberative reasoning style. However, people with ASD self-reported that they have difficulties making decisions quickly, which is consistent with the idea of an impairment in the rapid reasoning that characterises intuition.

It should be noted that the intuitive response is actually a wrong choice, despite being the dominant response in highly educated American college students (Frederick 2005). A propensity to engage in deliberative reasoning within a context that typically triggers erroneous intuitive reasoning can be seen as an advantage associated with ASD (and potentially higher autism traits). Toplak et al. (2011, 2014) provide an extensive analysis of the correlates of the CRT with other reasoning tasks, however there are no tasks which assess intuition independently of deliberation. Within the CRT, intuitive reasoning results in erroneous responses and could therefore be regarded as a deficit within this context that people with ASD do not demonstrate to the same degree as the general population.^1^ Toplak et al. (2011, 2014) argue that the CRT is a unique predictor of susceptibility to biases. As biases occur through the application of heuristics rather than engaging in further analytic processing, Toplak et al. describe the CRT as a particularly potent measure of ‘miserly’ cognitive processing (or ‘lazy thinking’, Kahneman 2011: 48). Defaulting to the reduced processing demands of being a cognitive miser has been argued to be typical in many contexts (cf. Fiske and Taylor 1991). From this perspective, ASD is associated with not being cognitively miserly (or lazy thinking), and the proposed reasoning bias is best characterised as being *unbiased* (within the context of the CRT at least).

Thus, in the present study, the ASD group obtained more correct answers than the TD group which is consistent with the literature identifying a diminished framing effect in ASD (De Martino et al. 2008). The deliberative score on the CRT relates to a wide range of rational reasoning tasks (Lesage et al. 2013; Sirota et al. 2014; Toplak et al. 2011, 2014). However, whilst self-reported intuition can negatively correlate with rational reasoning (Shiloh et al. 2002), apart from the intuitive subscale of the REI, assessments of intuition are rare (see Pennycook et al. 2015). The proposed advantages in a greater propensity towards deliberative reasoning in ASD may have an associated cost on intuitive reasoning. However, this was not independently evidenced in this study as we did not have a behavioural measure of intuition independent of deliberation. Recall also, the variation in findings regarding the relationship between behavioural and self-reported intuition in the general population identified in the literature, which may indicate that the intuitive subscale of the REI is not a reliable index of intuitive behaviour.

Despite issues with assessment, potential biases away from intuitive reasoning in other contexts, such as social contexts, may be pertinent to ASD as they may be associated with deficits in empathy (e.g. Baron-Cohen and Wheelwright 2004). The social world often requires rapid processing of social cues occurring in an uncertain context rather than an overt ‘rule-based’ system. Within their Enactive Mind approach, Klin et al. (2003) characterise the social world as an ‘open domain task’ requiring an understanding of the relative significance of a multitude of elements, the importance of which are dependent upon the context of the situation. Future research can develop the extent to which a propensity towards deliberative processing within naturalistic social settings is too slow and effortful to allow for effective participation. As Darius (2002: 25; cited in Davidson 2008) recounts: ‘There is no such thing as adequate delayed social reactions. One is either quick enough to keep up, or one is weird and socially disabled’. Rapidly and automatically extracting emotional information from social environments is argued to be an intuitive process that feeds ‘downstream’ empathy processes and related social–emotional functioning (Clark et al. 2008; Kahneman 2011; see also Rump et al. 2009; Tracy et al. 2011). Thus, the unbiased use of deliberation identified in the present research may relate to the social-emotional weaknesses which form part of the diagnostic criteria for ASD. Consistent with this, the measures on intuition used in the present study have been found to correlate with measures of empathy in a general population (Brosnan et al. 2014b). Dual Process Theory may therefore extend beyond reasoning to provide a fuller account of the social cognition that characterises ASD (see Evans 2008; Sherman et al. 2014).

Thus a bias towards deliberative reasoning within Dual Process Theory may provide an account of the strengths associated with ASD. Dual Process Theory therefore might usefully bring to bear additional cognitive research from non-clinical groups pertaining to how combinations of intuition and deliberation may relate to ASD. For example, the default-interventionist position could characterise the application of deliberative reasoning in ASD to typically intuitive tasks such as emotion recognition (‘corners of mouth turned down, lowered eyebrows = sad’: Rutherford and McIntosh 2007; Walsh et al. 2014; see also Brosnan et al. 2015b; Golan and Baron-Cohen 2006; Golan et al. 2010). Within Dual Process Theory, intuitive tasks such as rapid emotion recognition, would be expected to be independent of working memory and cognitive abilities in a general population, but not in an ASD group who were utilising deliberative reasoning (see Harms et al. 2010, for review of the evidence for this). Physically slowing stimuli would also be predicted to enhance the performance of those utilising deliberative reasoning strategies (see Gepner et al. 2001; Tardif et al. 2007; Gepner and Féron 2009). Typically, intuitive and deliberative reasoning can be applied as appropriate to the perceived demands of the reasoning context. The present study is consistent with the idea that those with ASD do not have the balance of reasoning styles but have a bias towards deliberative reasoning and away from intuitive reasoning across contexts. There may be contexts where this is beneficial (e.g. mathematics) and contexts where this is detrimental (e.g. social).

Interestingly, Freeman et al. (2012) also propose an imbalance in reasoning styles is related to schizotypy traits in a non-clinical population sample. Freeman et al. found that higher intuitive reasoning combined with lower deliberative reasoning was related to the degrees of schizotypy traits, which is the opposite pattern to that associated with autism traits in the present study. Those with high levels of schizotypy have also been found to bias towards making decisions rapidly (‘jumping to conclusions’; Freeman 2007; Freeman et al. 2008; Garety et al. 2005, 2007) again reflecting the opposing pattern to higher autism traits and ASD identified in the present study. Schizotypy was not assessed in the present study, but it is interesting to speculate that variation in the relative biases towards intuition and deliberation within Dual Process Theory may represent a framework within which similarities and differences between these clinical conditions and associated traits across the general population can be further explored (see Crespi and Badcock 2008; see also Brosnan et al. 2010; Chisholm et al. 2015 for review). Within the non-clinical population, the extent to which individuals engage in intuitive or deliberative reasoning has been found to be susceptible to manipulation. For example, being told to ‘think carefully’ or to write down details of how you came to a decision have been found to elicit more deliberative responses, where as being instructed to go with a ‘gut-feeling’ has been found to elicit more intuitive responses (e.g. Dijkstra et al. 2012; Usher et al. 2011). However, if reasoning in ASD is characterised by diminished intuitive mechanisms, whether such manipulations would affect those with ASD is an open question future research. Further, there is also the intriguing possibility that even if people with ASD were shown not to employ rapid, effortless, parallel, non-conscious processing (‘intuition’) in social contexts they may default to them in other contexts (e.g. there are alternative ‘open domain tasks’; Klin et al. 2003).

There were limitations to the present research. All of the participants were at university or intending to go to university and were therefore not reflective of either the ASD or non-clinical populations as a whole. This may relate to the lack of sex differences in Study One. The sample was also not specified in terms of further demographics, such as social economic status, which limits generalisability. The participants in Study 2 were all considering attending university, and the degree of ASD symptomology was likely mild and the findings may not extend to the whole autism spectrum. Study 2 only compared male participants which is another limitation of the study, especially given the relatively little understanding of female populations with ASD (Halladay et al. 2015). In addition, only 11 participants with ASD completed the REI in Study 2, and the potential for a Type 1 error needs to be borne in mind. Whilst the CRT is a widely used measure of intuitive and deliberative responding, it should be noted that the intuitive response is wrong. Other wrong responses are not considered intuitive and are not typically analysed (hence the task is not purely ipsative). Analysing errors may provide useful insights into whether they emerge from intuitive or deliberative reasoning (Brosnan et al. 2015a). In addition, the terms intuition and deliberation have been used for stylistic convenience, but it should be noted that they refer to two clusters of concepts (Type 1 and Type 2 respectively, see Evans 2008 for a review; see Keren and Schul 2009 for critique; Kruglanski and Gigerenzer 2011 for an alternative view) that share similarities, but also have differences, within each cluster. Finally, an independent assessment of IQ was not undertaken, which is a major limitation of the study. Whist intuitive reasoning is argued to be independent of cognitive abilities, deliberative reasoning is not. The participants were studying at the same educational level (A-levels, examinations typically taken at 18 years of age for University entry) though may not attain the same grades. Future research can address the assessment of cognitive ability. Although college students do not necessarily have higher levels of autism traits than random control groups, those studying sciences (including mathematics) have been shown to have higher levels of autism traits than those studying humanities and social sciences (Baron-Cohen et al. 2001a, b), which can also be explored in future research.

## Footnotes

1 We thank an anonymous reviewer for highlighting this point.

## References

[CR1] Allison C, Auyeung B, Baron-Cohen S (2012). Toward brief “Red Flags” for autism screening: The Short Autism Spectrum Quotient and the Short Quantitative Checklist for Autism in toddlers in 1,000 cases and 3,000 controls. Journal of the American Academy of Child and Adolescent Psychiatry.

[CR2] Allman JM, Watson KK, Tetreault NA, Hakeem AY (2005). Intuition and autism: A possible role for Von Economo neurons. Trends in Cognitive Sciences.

[CR3] American Psychiatric Association. (1994). *DSM IV*. American Psychiatric Association.

[CR4] American Psychiatric Association (2013). Diagnostic and statistical manual of mental disorders.

[CR5] Baron-Cohen S, Wheelwright C (2004). The Empathy Quotient (EQ). An investigation of adults with Asperger syndrome or high functioning autism, and normal sex differences. Journal of Autism and Developmental Disorders.

[CR6] Baron-Cohen S, Wheelwright S, Hill J, Raste Y, Plumb I (2001). The “Reading the Mind in the Eyes” test revised version: A study with normal adults, and adults with Asperger syndrome or high-functioning autism. Journal of Child Psychology and Psychiatry.

[CR7] Baron-Cohen S, Wheelwright S, Skinner R, Martin J, Clubley E (2001). The Autism-Spectrum Quotient (AQ): Evidence from Asperger syndrome/high-functioning autism, males and females, scientists and mathematicians. Journal of Autism and Developmental Disorders.

[CR8] Brosnan M, Ashwin C, Gamble T (2013). Greater empathizing and reduced systemizing in people who show a jumping to conclusions bias in the general population: Implications for psychosis. Psychosis.

[CR9] Brosnan M, Ashwin C, Walker I, Donaghue J (2010). Can an ‘Extreme Female Brain’ be characterised in terms of psychosis?. Personality and Individual Differences.

[CR10] Brosnan M, Chapman E, Ashwin C (2014). Adolescents with autism spectrum disorder show a circumspect reasoning bias rather than ‘jumping-to-conclusions’. Journal of Autism and Developmental Disorders.

[CR11] Brosnan M, Hollinworth M, Antoniadou K, Lewton M (2014). Is empathizing intuitive and systemizing deliberative?. Personality and Individual Differences.

[CR12] Brosnan, M., Johnson, H., Grawemeyer, B., Chapman, E., Antoniadou, K., & Hollinworth, M. (2015a). Deficits in metacognitive monitoring in mathematics assessments in learners with autism spectrum disorder. *Autism.* doi:10.1177/1362361315589477. 10.1177/1362361315589477 26101449

[CR13] Brosnan M, Johnson H, Grawmeyer B, Chapman E, Benton L (2015). Emotion recognition in animated compared to human stimuli in adolescents with autism spectrum disorder. Journal of Autism and Developmental Disorders.

[CR14] Cacioppo JT, Petty RE (1982). The need for cognition. Journal of Personality and Social Psychology.

[CR15] CDC. (2015). http://www.cdc.gov/mmwr/preview/mmwrhtml/ss6302a1.htm?s_cid=ss6302a1_w. Accessed June 17, 2015.

[CR16] Chisholm K, Lin A, Abu-Akel A, Wood SJ (2015). The association between autism and schizophrenia spectrum disorders: A review of eight alternate models of co-occurrence. Neuroscience and Biobehavioral Reviews.

[CR17] Clark TF, Winkielman P, McIntosh DN (2008). Autism and the extraction of emotion from briefly presented facial expressions: Stumbling at the first step of empathy. Emotion.

[CR18] Constantino J, Todd R (2003). Autistic traits in the general population—a twin study. Archives of General Psychiatry.

[CR19] Crespi B, Badcock C (2008). Psychosis and autism as diametrical disorders of the social brain. Behavioral and Brain Sciences.

[CR20] Darius. (2002). Darius’s Essay. In D. Prince-Hughes (ed.), *Aquamarine Blue 5: Personal stories of college students with autism* (pp. 9–42). Athens, OH: Swallow Press.

[CR21] Davidson J (2008). Autistic culture online: Virtual communication and cultural expression on the spectrum. Social and Cultural Geography.

[CR22] De Martino B, Harrison NA, Knafo S, Bird G, Dolan RJ (2008). Explaining enhanced logical consistency during decision making in autism. The Journal of Neuroscience.

[CR23] Dijkstra KA, van der Pligt J, van Kleef GA, Kerstholt JH (2012). Deliberation versus intuition: Global versus local processing in judgment and choice. Journal of Experimental Social Psychology.

[CR24] Epstein S, Pacini R, Denes-Raj V, Heier H (1996). Individual differences in intuitive–experiential and analytical–rational thinking styles. Journal of Personality and Social Psychology.

[CR25] Evans JSB (2008). Dual-processing accounts of reasoning, judgment, and social cognition. Annual Review of Psychology.

[CR26] Evans JSB (2011). Dual-process theories of reasoning: Contemporary issues and developmental applications. Developmental Review.

[CR27] Evans JSB, Curtis-Holmes J (2005). Rapid responding increases belief bias: Evidence for the dual-process theory of reasoning. Thinking & Reasoning.

[CR28] Evans JSB, Frankish KE (2009). In two minds: Dual processes and beyond.

[CR29] Evans JSB, Stanovich KE (2013). Dual-process theories of higher cognition advancing the debate. Perspectives on Psychological Science.

[CR30] Fiske ST, Taylor SE (1991). Social cognition.

[CR31] Frederick S (2005). Cognitive reflection and decision making. Journal of Economic Perspectives.

[CR32] Freeman D (2007). Suspicious minds: The psychology of persecutory delusions. Clinical Psychology Review.

[CR33] Freeman D, Evans N, Lister R (2012). Gut feelings, deliberative thought, and paranoid ideation: A study of experiential and rational reasoning. Psychiatry Research.

[CR34] Freeman D, Pugh K, Garety P (2008). Jumping-to-conclusions and paranoid ideation in the general population. Schizophrenia Research.

[CR35] Garety PA, Bebbington P, Fowler D, Freeman D, Kuipers E (2007). Implications for neurobiological research of cognitive models of psychosis. Psychological Medicine.

[CR36] Garety PA, Freeman D, Jolley S, Dunn G, Bebbington PE, Fowler D (2005). Reasoning, emotions and delusional conviction in psychosis. Journal of Abnormal Child Psychology.

[CR37] Gepner B, Deruelle C, Grynfeltt S (2001). Motion and emotion: A novel approach to the study of face processing by young autistic children. Journal of Autism and Developmental Disorders.

[CR38] Gepner B, Féron F (2009). Autism: A world changing too fast for a mis-wired brain?. Neuroscience and Biobehavioral Reviews.

[CR39] Golan O, Ashwin E, Granader Y, McClintock S, Day K, Leggett V (2010). Enhancing emotion recognition in children with autism spectrum conditions: An intervention using animated vehicles with real emotional faces. Journal of Autism and Developmental Disorders.

[CR40] Golan O, Baron-Cohen S (2006). Systemizing empathy: Teaching adults with Asperger syndrome or high-functioning autism to recognize complex emotions using interactive multimedia. Development and Psychopathology.

[CR41] Halladay AK, Bishop S, Constantino JN, Daniels AM, Koenig K, Palmer K (2015). Sex and gender differences in autism spectrum disorder: Summarizing evidence gaps and identifying emerging areas of priority. Molecular Autism.

[CR42] Harms MB, Martin A, Wallace GL (2010). Facial emotion recognition in autism spectrum disorders: A review of behavioral and neuroimaging studies. Neuropsychology Review.

[CR43] Kahneman D (2011). Thinking, fast and slow.

[CR44] Keren G, Schul Y (2009). Two is not always better than one a critical evaluation of two-system theories. Perspectives on Psychological Science.

[CR45] Klin A, Jones W, Schultz R, Volkmar F (2003). The enactive mind, or from actions to cognition: Lessons from autism. Philosophical Transactions of the Royal Society B: Biological Sciences.

[CR46] Klin A, Volkmar FR, Cohen DJ, Volkmar FR (1997). Asperger’s syndrome. Handbook of autism and pervasive developmental disorders.

[CR47] Kruglanski AW, Gigerenzer G (2011). Intuitive and deliberate judgments are based on common principles. Psychological Review.

[CR48] Lesage E, Navarrete G, De Neys W (2013). Evolutionary modules and Bayesian facilitation: The role of general cognitive resources. Thinking & Reasoning.

[CR49] Liberali JM, Reyna VF, Furlan S, Stein LM, Pardo ST (2012). Individual differences in numeracy and cognitive reflection, with implications for biases and fallacies in probability judgment. Journal of Behavioural Decision Making.

[CR50] Luke L, Clare ICH, Ring H, Redley M, Watson P (2012). Decision-making difficulties experienced by adults with autism spectrum conditions. Autism.

[CR51] Pacini R, Epstein S (1999). The relation of rational and experiential information processing styles to personality, basic beliefs, and the ratio-bias phenomenon. Journal of Personality and Social Psychology.

[CR52] Pennycook, G., Cheyne, J. A., Koehler, D. J., & Fugelsang, J. A. (2015). Is the cognitive reflection test a measure of both reflection and intuition? *Behavior Research Methods*. doi:10.3758/s13428-015-0576-1. 10.3758/s13428-015-0576-1 25740762

[CR53] Plomin R, Haworth CMA, Davis OSP (2009). Common disorders are quantitative traits. Nature Reviews Genetics.

[CR54] Posserud MB, Lundervold AJ, Gillberg C (2006). Autistic features in a total population of 7–9-year-old children assessed by the ASSQ (Autism Spectrum Screening Questionnaire). Journal of Child Psychology and Psychiatry.

[CR55] Ritvo RA, Ritvo ER, Guthrie D, Ritvo MJ, Hufnagel DH, McMahon W (2011). The Ritvo Autism Asperger diagnostic scale-revised (RAADS-R): A scale to assist the diagnosis of autism spectrum disorder in adults: An international validation study. Journal of Autism and Developmental Disorders.

[CR56] Rump KM, Giovannelli JL, Minshew NJ, Strauss MS (2009). The development of emotion recognition in individuals with autism. Child Development.

[CR57] Rutherford MD, McIntosh DN (2007). Rules versus prototype matching: Strategies of perception of emotional facial expressions in the autism spectrum. Journal of Autism and Developmental Disorders.

[CR58] Rutter, M., Bailey, A., & Lord, C. (2003). *Social Communication Questionnaire (SCQ*). Western Psychological Services.

[CR59] Ruzich E, Allison C, Smith P, Watson P, Auyeung B, Ring H (2015). Measuring autistic traits in the general population: A systematic review of the Autism-Spectrum Quotient (AQ) in a nonclinical population sample of 6,900 typical adult males and females. Molecular Autism.

[CR60] Sherman JW, Gawronski B, Trope Y (2014). Dual-process theories of the social mind.

[CR61] Shiloh S, Salton E, Sharabi D (2002). Individual differences in rational and intuitive thinking styles as predictors of heuristic responses and framing effects. Personality and Individual Differences.

[CR62] Sirota M, Juanchich M, Hagmayer Y (2014). Ecological rationality or nested sets? Individual differences in cognitive processing predict Bayesian reasoning. Psychonomic Bulletin Review.

[CR63] Stanovich KE, West RF (2000). Individual differences in reasoning: Implications for the rationality debate?. Behavioral and Brain Sciences.

[CR64] Stanovich KE, West RF (2008). On the relative independence of thinking biases and cognitive ability. Journal of Personality and Social Psychology.

[CR65] Tardif C, Lainé F, Rodriguez M, Gepner B (2007). Slowing down presentation of facial movements and vocal sounds enhances facial expression recognition and induces facial–vocal imitation in children with autism. Journal of Autism and Developmental Disorders.

[CR66] Thoma V, White E, Panigrahi A, Strowger V, Anderson I (2015). Good thinking or gut feeling? Cognitive reflection and intuition in traders, bankers and financial non-experts. PLoS ONE.

[CR67] Toplak ME, West RF, Stanovich KE (2011). The cognitive reflection test as a predictor of performance on heuristics and biases tasks. Memory & Cognition.

[CR68] Toplak ME, West RF, Stanovich KE (2014). Assessing miserly information processing: An expansion of the cognitive reflection test. Thinking & Reasoning.

[CR69] Tracy JL, Robins RW, Schriber RA, Solomon M (2011). Is emotion recognition impaired in individuals with autism spectrum disorders?. Journal of Autism and Developmental Disorders.

[CR70] Tversky A, Kahneman D (1974). Judgment under uncertainty: Heuristics and biases. Science.

[CR71] Usher M, Russo Z, Weyers M, Brauner R, Zakay D (2011). The impact of the mode of thought in complex decisions: Intuitive decisions are better. Frontiers in Psychology.

[CR72] Walsh JA, Vida MD, Rutherford MD (2014). Strategies for perceiving facial expressions in adults with autism spectrum disorder. Journal of Autism and Developmental Disorders.

[CR73] Wing L, Wing L (1988). The autistic continuum. Aspects of autism: Biological research.

[CR74] Witwer AN, Lecavalier L (2008). Examining the validity of autism spectrum disorder subtypes. Journal of Autism and Developmental Disorders.

[CR75] World Health Organization (1992). The ICD-10 classification of mental and behavioural disorders: Clinical descriptions and diagnostic guidelines.

